# Supplementary material for: Cardiac Functional and Structural Abnormalities in a Mouse Model of CDKL5 Deficiency Disorder
Source: Int J Mol Sci. 2023 Mar 14;24(6):5552. doi: 10.3390/ijms24065552 (PMC10059787; doi:10.3390/ijms24065552)
Supplement: Supplementary file 1 [file ijms-24-05552-s001.zip › ijms-2239008-supplementary.pdf]

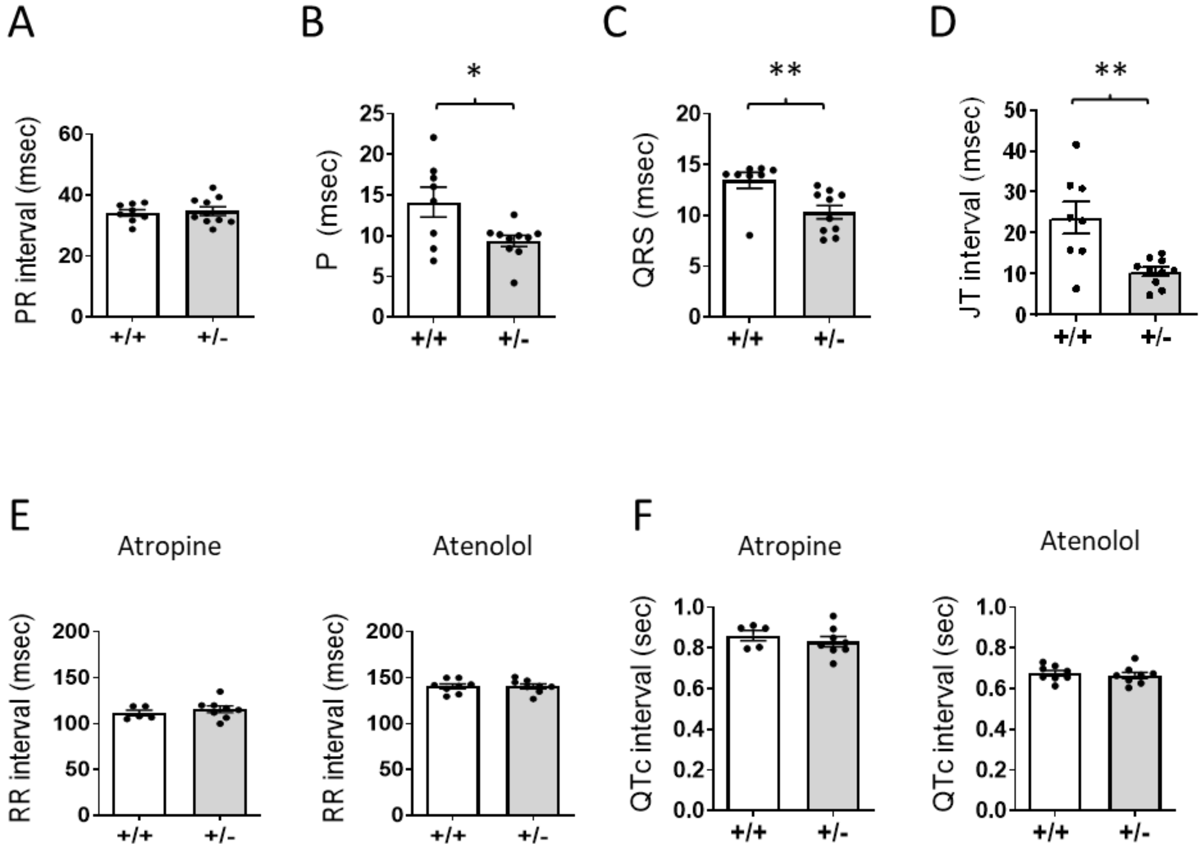

Supplementary Figure S1.

A-D: Baseline ECG parameters calculated during non-rapid-eye-movement sleep (NREMS) in *Cdkl5* +/+ (n=8) and *Cdkl5* +/- (n=10) female mice. **A:** Mean interval between the beginning of the P wave (onset of atrial depolarization) and the beginning of the QRS complex (onset of ventricular depolarization). **B:** Mean duration of atrial depolarization (P wave). **C:** Mean duration of the depolarization of the right and left ventricles (QRS complex). **D:** Mean duration of ventricular repolarization (JT interval). **E,F**: Absolute value of RR (mean heart period duration, E) and QTc (mean duration of interval between Q and T waves after applying Hodge's formula, F) intervals after either Atropine (*Cdkl5 +/+* n= 5 and *Cdkl5 +/-* n= 8) or Atenolol (*Cdkl5 +/+* n= 8 and *Cdkl5 +/-* n= 8) infusion. Results are presented as means ± SEM. * $p<0.05$, ** $p<0.01$; (two-tailed Student's t-test after 2-way ANOVA).

Gene		Primer sequence (5’-3’)
Cdkl5	Forward	TGCAGACACAAGGAAACACATGA
	Reverse	TTTCCTGCTTGAGAGTGCGAA
Gapdh	Forward	CCAGTGAGCTTCCCGTTCA
	Reverse	GAACATCATCCCTGCATCCA
Actb	Forward	AAGTGGTTACAGGAAGTCC
	Reverse	ATAATTTACACAGAAGCAATGC
Kcnq1	Forward	ATCGGTGCCCGTCTGAACAG
	Reverse	TTGCTGGGTAGGAAGAGCTCA
Kcnh2	Forward	GATCGCCTTCTACCGGAAA
	Reverse	CATTCTTCACGGGTACCACA
Kcnj2	Forward	CCCCATGATCCTGTACCAG
	Reverse	ATGGATGCTTCCGAGAACC
Scn5a	Forward	GCAGAAGGTGAAGTTCGTGG
	Reverse	TGAAGACCAAGTTTCCGACC
Hcn4	Forward	CGACAGCGCATCCATGACTA
	Reverse	GCTGGAAGACCTCGAAACGC
Chrm2	Forward	CCCCAATACAGTGTGGACAA
	Reverse	GCAGGGTTGATGGTGCTATT
Adrb1	Forward	GCTCTGGACTTCGGTAGATGTG
	Reverse	CGTCAGCAAACTCTGGTAGCGA
Col1a2	Forward	CACCCCAGCGAAGAACTCAT
	Reverse	TCTCCTCATCCAGGTACGCA
Col3a1	Forward	TGACTGTCCCACGTAAGCAC
	Reverse	AGGGCCATAGCTGAACTGAA
Gja1	Forward	GAGAGCCCGAACTCTCCTTT
	Reverse	TGGGCACCTCTCTTTCACTT

Supplementary Table S1. List of primers used for quantitative RT-PCR.

Antibody against	Description	Use/Diluition	Product nr and Manufacturer
Actinin	Goat polyclonal	IHC 1:100	17829, Santa Cruz Biotechnology
AKT	Rabbit polyclonal	WB 1:1000	4691, Cell Signaling Technology
β-Catenin	Rabbit polyclonal	WB 1:500<br>IHC 1:300	05665, Millipore
Cx43	Rabbit monoclonal	WB 1:500<br>IHC 1:200	3512, Cell Signaling Technology
ERK1/2	Rabbit polyclonal	WB 1:1000	4695, Cell Signaling Technology
GAPDH	Rabbit polyclonal	WB 1:5000	G9545, Sigma-Aldrich
GSK-3β	Rabbit polyclonal	WB 1:1000	9315, Cell Signaling Technology
LC3B	Rabbit polyclonal	WB 1:1000	PA1-16930, Thermo Fisher Scientific
Nrf 2	Rabbit polyclonal	WB 1:500	365949, Santa Cruz Biotechnology
P-AKT (Ser473)	Rabbit polyclonal	WB 1:1000	4060, Cell Signaling Technology
P-ERK1/2 (Thr202/Tyr204)	Rabbit polyclonal	WB 1:1000	9101, Cell Signaling Technology
P-GSK-3β (Ser9)	Rabbit polyclonal	WB 1:1000	5558, Cell Signaling Technology
PARP1	Rabbit polyclonal	WB 1:500	227244, Abcam
Vimentin	Mouse monoclonal	WB 1:500	V6630, Sigma-Aldrich

Secondary antibodies

Antibody	Conjugate	Use/Diluition	Product nr and Manufacturer
Donkey Anti-Rabbit IgG	Cy3	IHC 1:200	711-165-152, Jackson ImmunoResearch Laboratories, Inc.
Donkey Anti-Goat IgG	Cy3	WB 1:200	705-165-147, Jackson ImmunoResearch Laboratories, Inc.
Goat Anti-Mouse IgG	Cy3	IHC 1:200	115-165-062, Jackson ImmunoResearch Laboratories, Inc.
Goat Anti-Mouse IgG	HRP	WB 1:5000	115-005-003, Jackson ImmunoResearch Laboratories, Inc.
Goat Anti-Rabbit IgG	HRP	WB 1:5000	111-035-003, Jackson ImmunoResearch Laboratories, Inc.

Supplementary Table S2 *WB, western blot; IHC, Immunohistochemistry
